# DECORE-21: Assessment of occupational stress in police. Confirmatory factor analysis of the original model

**DOI:** 10.1371/journal.pone.0205028

**Published:** 2018-10-04

**Authors:** Beatriz Talavera-Velasco, Lourdes Luceño-Moreno, Jesús Martín García, Daniel Vázquez-Estévez

**Affiliations:** Department of Social, Organizational and Differential Psychology, Faculty of Psychology, Complutense University of Madrid, Madrid, Spain; Universidad Miguel Hernandez de Elche, SPAIN

## Abstract

There is scarce research on stress in Spanish police officers and on the use of instruments to assess this construct in this professional group. In Spain, the DECORE questionnaire has been one of the most used. Nevertheless, it had not been previously applied to police officers. The aim of this study was to analyze both the construct validity and the reliability of the original model of 44 items. A cross-sectional design and a non-probabilistic quota sampling were used. A total of 223 Spanish police officers participated. 202 police personnel were men (90.6%) and 21 were women (9.4%). The average age was 41 years old (SD = 7.52). These police officers answered the DECORE questionnaire. Confirmatory Factor Analysis was carried out. The results showed an unsatisfactory adjustment using the original model of 44 items. A final solution of 21 items and four factors was formulated with both suitable validity and reliability indexes. In this model, 23 items that did not weigh highly in any of the four factors were removed. The DECORE-21 questionnaire is recommended to assess work-related stress in police officers.

## Introduction

The number of research articles focusing on psychosocial risk factors at work has increased in the last fifteen years, due to the relationship with occupational stress [1]. The European Commission defines the term occupational stress as the “set of emotional, cognitive, physiological and behavioral reactions to harmful aspects of the content, organization or context of the work. It is a condition that is characterized by high levels of excitation, distress and the feeling of not being able to do anything” [2]. Psychosocial risk factors at work can negatively affect the health of the worker, producing stress [3]. They are defined as “those conditions that are present in a labor situation and that are directly related to the organization, the content of the work and the accomplishment of the task, and that have aptitude to concern so much the well-being or the health (physical, psychological or social) of the worker as the development of the work” [4]. Examples of psychosocial risk factors at work are the lack of control during tasks, unforeseeable work schedules, social isolation, labor uncertainty or the perception of high job demands. Specifically, the perception of: lack of control of work, interpersonal conflicts, perception of high job demands (like the increase of both work hours and workload), or the perception of low organizational support are some of the psychosocial risk factors at work related to cardiovascular problems [5], anxiety, depression [6] or musculoskeletal disorders [7].

There are different instruments that assess the psychosocial risk factors at work such as questionnaires, inventories or scales, check-lists, administrative information, interviews, discussion groups (or focus-group) and the information contained in the job description. Questionnaires have been widely used because of: their anonymous character, easiness to fill in, standardized characteristics and the fact that they allow us to obtain an important quantity of information in little time, making them very practical tools [3].

The questionnaires most used for the assessment of the stress at work that appear in the scientific bibliography are the following ones: a) Job Content Questionnaire-JCQ [8]; b) Effort-Reward Imbalance Questionnaire-ERI-Q [9]; and, c) Job Stress Survey-JSS [10]. In Spain, the DECORE questionnaire to assess the psychosocial risks has shown evidences of validity and reliability [11]. However, it is necessary to apply the questionnaire in other job positions, different from the ones used in the original adaptation of the questionnaire. In addition, it is advisable to examine the psychometric properties of the instruments of evaluation in different samples throughout the time [12]. The DECORE questionnaire has been applied in military personnel but not in other security forces such as the police.

In general, the police profession is characterized for being a job position of high risk for the development of occupational stress. Police officers must assist citizens and carry out monotonous tasks. Usually they feel stressed out and they feel that they are overloaded in their work [13]. Most of the stressors perceived by police officers come from both the organization and their tasks [14], so there is a high probability that these workers suffer from work-related stress and health problems [15]. Some tasks, such as, mediating in conflicts between citizens, directing traffic, reporting infractions, enforcing the law, attending to citizens or pursuing organized crime, produce high levels of stress in police officers [16, 17].

Organizational support has been one of the factors that have best explained stress in police officers. For these workers, the support received from colleagues and supervisors is essential [18]. Other factors such as the perception of low control over labor demands in different situations make the work of the police highly stressful [19], and consequently they have a greater probability of suffering from cardiovascular diseases [20] and poor mental health [21].

Only one study has been found that treats the differences in stress between local police officers according to gender, seniority or type of work-shift in Spain [22]. Therefore, it is necessary to carry out studies on the assessment of stress in this profession. Nevertheless, it is true that both the access to this type of sample and the assessment of the psychosocial risk factors are difficult, due to the reticence of some members of the organization [23]. For this reason, it becomes necessary to perform more precise evaluations, reduce their duration and develop specific instruments for assessment in this profession.

Therefore, the aim of this research was to validate the DECORE questionnaire in a sample of police officers, with the intention of assuring that its psychometric properties are adequate, and its application is useful for this professional group.

## Materials and methods

### Participants

For this research, a cross-sectional design and a non-probabilistic quota sampling was used. This type of sampling means that, despite all the police officers who participated did so voluntarily, it was necessary that they meet several requirements. Those officers who had at one year seniority at least in the work place at and the same police station were selected. The total sample consisted of 223 police officers from Madrid, Spain. Of the total number of participants, 202 were men (90.6%) and 21 women (9.4%). The average age was 41 years old (SD = 7.52). The average of job seniority was 14 years (SD = 13.90). The average work hours per week was 38 hours (SD = 8.87). Regarding the type or shift, 36.8% of the total number worked in the morning shift; 27.4% had the afternoon shift; 23.8% worked in the night shift; finally, 12% had a rotating shift. All the participants were local police officers.

### Instrument

To assess the perception of the psychosocial risk factors at work, the DECORE Questionnaire for the Psychosocial Risk Assessment [11] was applied. This questionnaire assesses the perception that the workers have on the following psychosocial risk factors: excess (or shortage) of cognitive demands, lack of control, little organizational support and lack of rewards. The questionnaire consists of 44 items and the following scales: a) Cognitive demands (12 items): it refers to the requirements that workers must meet, which are related to the perception of the quantitative workload that the worker carries out. Examples of items of this scale are: "I feel a lot of time pressure to do my job" or "I work more hours than necessary"; b) Control (9 items): the items of this scale assess the possibilities that the worker has so that he can determine both the methods of organization of work and the decisions related to the organization of work activities. Examples of items: "I can interrupt my work if necessary" or "I have little freedom to decide the way of doing the work"; c) Organizational support (12 items): it assesses the perception of the workers about the relationships that they have with colleagues and supervisors. Examples of items of this scale would be: "Relations with my coworkers are good" or "In my work I have easy access to colleagues and superiors"; and, finally, d) Rewards (11 items): The items of this scale assess the benefits that the worker perceives due to the performance of his/her tasks (specially, it assesses the perception of salary and job safety). Examples of items would be: "I think that the pay policy of my company is adequate" or "The future prospects of an increase in salary are good". Apart from the scoring on every scale, it is possible to calculate the Global Risk Index (IGR). This index offers an overall scoring of the perception of psychosocial risk, considering the average of the scores of the four dimensions. All the items are answered on a five-point Likert scale, ranging from 1 (strongly disagree) to 5 (strongly agree). A high score indicates a very adverse situation from the point of view of the psychosocial risk (the worker perceives excessive cognitive demands, lack of control, scarce organizational support and few rewards in his/her work). The range of scores for each of the scales, as well as for the global scale, ranges between 100 and 500. A high score indicates that the worker perceives the psychosocial risk factors adversely. The validity of the questionnaire was analyzed by factor analysis, resulting in a structure made up of four factors. The coefficients Cronbach’s alpha are ranged between .81 and. 84, so the questionnaire is considered as an instrument that presents suitable evidence of validity and reliability [22, 24]. Nevertheless, though the psychometric properties of the questionnaire are robust in other samples, it has not been applied previously in police officers.

### Procedure

This study was approved by the Ethics Committee of the Faculty of Psychology of the Complutense University of Madrid, obtaining a favorable report on February 2017 (Ref. 2016/17-012). Representatives of workers and local police departments were informed of the nature of this research and its voluntary and anonymous nature. A collaborative agreement was signed between the police corps and the Complutense University of Madrid. In this agreement, special attention was paid to the preservation of anonymity and the objective of using the data with a purely scientific and investigative purpose was specified, without prejudice to the participating police officers or to the organization. The policemen completed the questionnaire and signed an informed consent. The questionnaires were administered in paper form and gathered in closed envelope for each participant. 225 questionnaires were completed, but two police officers had to be eliminated from the sample because they answered less than 50% of the items.

### Data analysis

The data analyses were carried out with the Lavaan package [25] of the programming environment R [26], using the R Studio interface [27] and the statistical package SPSS for Windows 21.0 [28]. The psychometric properties of the original model of 44 items of the DECORE questionnaire were analyzed. Construct validity and reliability analyses were carried out. A Confirmatory Factor Analysis (CFA) was conducted, since the theoretical model was already specified [29]. The criterion of Brown (2006) was used to assess the results [30]. This criterion recommends using CFI (Comparative Fit Index), TLI (Tucker-Lewis Index), RMSEA (Root Mean Square Error of Approximation) and SRMR (Standardized Root Mean Square Residual) indices to calculate both the satisfactory global functioning and model adjustment. Different indices of reliability were calculated: Cronbach’s alpha and McDonald’s Omega [31, 32].

## Results

### Construct validity

Regarding this type of validity, the indices CFI, TLI, RMSEA and SRMR were analyzed to assess the adjustment of the original model of 44 items in this sample (Table 1).

**Table 1:** Indices of adjustment obtained in the Confirmatory factor analysis using the DECORE-44 model.

Indices of adjustment	DECORE (44 items)	Accepted values
CFI	.82	≥ .90
TLI	.80	≥ .90
RMSEA	.08	≤ .05-.08
SRMR	.12	≤ .08

Note. CFI = Comparative Fit Index; TLI = Tucker-Lewis Index; RMSEA = Root Mean Square Error of Approximation; SRMR = Standardized Root Mean Square Residual.

Since it can be observed in Table 1, the fit indices CFI, TLI and SRMR for the original model of 44 items grouped in four factors (cognitive demands, control, organizational support and rewards) were below acceptable values (.82, .80, and .12, respectively). Only RMSEA remained adequate (.08).

At this point, the original model of DECORE was examined deeply. In this study, we detected items that did not seem to work the same way as on the original structural model, since they did not weigh highly in any of four factors. Therefore, we checked the factorial load of all the items of the model of 44 items. The obtained results can be observed in the Table 2. A conservative criterion was used, and items whose weight was lower than .60 were eliminated (Table 2). Items were eliminated depending on: the factorial loads, indices of modification, distribution analysis, reliability analysis as long as the content validity were not harmed.

**Table 2:** Item load corresponding to the original model of 44 items of the DECORE questionnaire in the sample of study, mean (*M*) and standard deviation (*SD*) by item.

Item	Factor					
	Cognitive demands	Control	Organizational Support	Rewards	*M*	*SD*
Cog1	.39				2.61	.96
Cog2	.10				1.58	.77
Cog3	.46				2.70	1.00
Cog4	.34				2.11	.95
Cog5	.58				2.96	1.10
Cog6	.65				3.70	1.03
Cog7	.74				3.74	.96
Cog8	.73				4.12	.72
Cog9	.55				3.94	.84
Cog10	.59				4.09	.84
Cog11	.74				4.31	.80
Cog12	.45				3.46	1.00
Con1		.55			3.17	1.05
Con2		.75			3.38	1.01
Con3		.73			3.09	1.19
Con4		.69			2.88	1.13
Con5		-.51			2.57	1.07
Con6		.59			2.94	1.10
Con7		- .70			2.74	1.11
Con8		- .45			1.99	.74
Con9		- .59			2.83	1.08
Org1			.79		2.76	1.25
Org2			.77		2.32	1.18
Org3			.56		4.08	.73
Org4			.78		3.75	.82
Org5			.64		3.79	.80
Org6			.59		3.70	.85
Org7			.53		3.37	.92
Org8			- .53		2.16	.80
Org9			- .43		1.65	.77
Org10			- .58		2.28	.72
Org11			.69		3.35	.96
Org12			- .63		3.08	1.07
Rew1				.87	2.89	1.11
Rew2				.79	2.39	1.04
Rew3				.69	1.91	.92
Rew4				.91	2.75	1.12
Rew5				.57	2.06	1.11
Rew6				.70	2.01	.91
Rew7				.51	2.74	1.01
Rew8				- .80	2.77	1.04
Rew9				.47	1.51	.62
Rew10				.52	1.57	.73
Rew11				.94	2.81	1.14

In total, 23 items were eliminated: 8 items of the Cognitive Demands factor; 5 items of the Control factor; 6 items of the Organizational Support factor; and, finally, 4 items of the Rewards dimension. After the elimination of the previous items, another CFA was carried out using the same fit indices. An adequate adjustment was found with the model of four factors and 21 items in the sample of this study (Table 3).

**Table 3:** Indices of adjustment obtained in the Confirmatory factor analysis using the DECORE-21 model.

Indices of adjustment	DECORE (21 items)	Accepted values
CFI	.95	≥ .90
TLI	.95	≥ .90
RMSEA	.07	≤ .05-.08
SRMR	.08	≤ .08

Note. CFI = Comparative Fit Index; TLI = Tucker-Lewis Index; RMSEA = Root Mean Square Error of Approximation; SRMR = Standardized Root Mean Square Residual.

As it can be observed in Table 3, by eliminating 23 items, a suitable adjustment of the model is obtained keeping its four dimensions. The Cognitive demands scale is now made up of 4 items, the same as the Control scale; the Organizational support scale consists of 6 items and the Rewards scale consists of 7 items.

The correlations, averages and standard deviations for each factor (cognitive demands, control, organizational support and rewards) are shown in Table 4.

**Table 4:** Correlations between factors, mean (*M*) and standard deviation (*SD*) of each dimension of DECORE-21 (*N* = 223).

	1	2	3	4
1.Cognitive demands	-	.24**	.16*	.22**
2.Control		-	.53**	.37**
3.Organizational support			-	.33**
4.Rewards				-
*M*	396.86	284.87	284.98	342.99
*SD*	67.21	84.01	72.16	81.89

Note.

*p < .05,

** *p* < .01.Factor

Table 4 shows the correlations between the different factors. A lowest value of .16 is obtained (p < .05) between cognitive demands and organizational support dimensions. The maximum value of .53 (p < .01) is reached between the control and organizational support factors. The averages in the different dimensions reveal values closer to 500 in all factors, the ones obtained in the cognitive demands (396.86) and rewards (342.99) dimensions being the highest averages.

### Reliability

The indices of reliability were calculated in both versions of the questionnaire (44 and 21 items) (Table 5).

**Table 5:** Reliability indices of the factors of DECORE-44 and DECORE-21.

	DECORE-44		DECORE-21	
Factors	α	Ω	α	Ω
Cognitive demands	.85	.85	.60	.67
Control	.83	.83	.78	.80
Organizational support	.88	.88	.84	.84
Rewards	.73	.76	.92	.92

Note. α: Cronbach´s alpha index; Ω: Omega index.

As we can see in Table 5, using the Cronbach’s alpha index, the levels of reliability are between .73 and .85 for all the scales of DECORE-44. The values of the omega index are similar to those of Cronbach’s alpha, with values between .76 and .88 in the scales of DECORE-44. With the new model of 21 items, the reliability indices are similar to the original model of 44 items. Only the Cognitive demands scale shows a value of .60 using alpha, although it comes closer to .70, using the omega index.

As a final result, the items of DECORE-21 questionnaire can be viewed in the Table 6.

**Table 6:** Items of the DECORE-21 questionnaire.

Factors	Ítems
Cognitive Demands	My job requires using complex or highly specialized skills (COG6).
	My job requires a high level of mental effort and full attention (COG7).
	My job requires collaborating different kinds of knowledge (COG8).
	The consequences of my mistakes are serious; therefore, my tasks at work involve a high degree of responsibility (COG11).
Control	If necessary, I can easily take a break (CON2).
	I can easily be a few minutes away of my work (5 or 10 minutes) (CON3).
	I can interrupt my work if necessary (CON4).
	In my job, I cannot choose when I take a holiday or when I can take days off (CON7).
Organizational Support	My bosses help me if I have problems with my job (ORG1).
	My bosses help me if I have personal problems not related to my job (ORG2).
	In general, there are good relationships at my place of work (ORG4).
	In my work I have easy access to colleagues and superiors (ORG5).
	My bosses and colleagues have a positive attitude towards my work (ORG11).
	The other departments do not usually help enough (ORG12).
Rewards	I think my salary is fair (REW1).
	I think that the pay policy of my company is adequate (REW2).
	The future prospects of an increase in salary are good (REW3).
	I am satisfied with my salary (REW4).
	Workers we enjoy enough benefits as we belong to the organization (REW6).
	I do not earn much money despite the effort I put in (REW8).
	I believe the money I earn for my job is fair enough (REW11).

## Discussion

The aim of this study was to assess the psychometric properties of the original version of 44 items of the DECORE questionnaire in a sample of Spanish police officers. The CFA showed an unsuitable fit. The analyses carried out indicated that some items presented low factorial weights. A conservative criterion was adopted, since it was considered necessary to eliminate the greater number of items that did not show adequate factorial weights, in order to shorten the time of application of the questionnaire in a sample of difficult access.

It is important to mention that, of the total number of 23 eliminated items, three of them presented values closer to .60: "I can decide the order in which I do my work", "I do not have any flexibility in organizing my work schedules" and "My work is demanding from an emotional point of view, due, for example, to contact with patients, clients, students, etc.". Specifically, the factorial loads of these items were: .596, .593 and .595, respectively. These three items should be analyzed in other studies to assess if they have a more satisfactory functioning in other samples. In this research, it was decided not to work with these three items because with 21 items the adjustment was satisfactory. Also, a minimum of four items were obtained by factor or dimension. Therefore, suitable psychometric properties are maintained with 21 items, and the use of this questionnaire is recommended in the police group. In addition, two indices of reliability were calculated. Commonly, Cronbach’s alpha index is used, though this index can underestimate the reliability in some cases. The omega index complements the information and can be considered useful, although its use is recommended with wide samples (N > 700).

This instrument could be used along other tools for measurement of burnout and symptoms associated with job stress in police officers. In Spain, the Burnout Granada’s Questionnaire was developed specifically for national police officers [33]. This questionnaire is composed by 26 items that assess the three factors for burnout. This one has been the only questionnaire that specializes in Spanish police officers. In comparison with the latter questionnaire, the DECORE-21 questionnaire presents a less number of items, evaluating the psychosocial risk factors (not the burnout construct), which facilitates the detection of variables related to stress that are susceptible to being manipulated by the organization in order to avoid development of chronic stress.

A greater number of studies on stress in police officers are necessary because of the difficulty of accessing these types of samples in the Public Administration [23]. Only ten studies with samples from police officers have been published in Spain in the last four years, although some of these studies investigated the transformational leadership in police, not stress [34]. Thus, it is necessary to consider that the psychosocial risk factors precede the appearance of chronic occupational stress and interventions can be designed to reduce the perceived stress. The Demand-Control model has been applied in samples of police officers in relation to the burnout syndrome, concluding that the perception of high job demands and scarce control over work environments are associated with emotional exhaustion, low perception of professional auto-efficacy and high levels of depersonalization [35, 36].

One of the principal contributions of this work is the validation of the DECORE-21 questionnaire for the assessment of the psychosocial risk factors at work in policemen. This version shortens the time for completing all the items, considering that the psychometric properties are improved. Comparing both versions of DECORE, the time for filling out the model of 21 items is reduced to approximately 25 minutes.

In other countries that are different than Spain, occupational stress has been evaluated specifically in police officers with the Police Stress Questionnaire [37]. This instrument consists of two scales (each of 20 items) designed to assess operational (PSQ-Op) and organizational (PSQ-Org) stress. The PSQ-Op measures different stressors, for example, fatigue and health problems related to work. The PSQ-Org measures stressors such as difficulties related to with lack of resources, red tape and staff shortages. The questionnaire is composed by 40 items [38]. In this regard, DECORE-21 shortens the time to fill it in and it includes the principal psychosocial risk factors that turn out to be more related to stress and disease in the bibliography. DECORE-21 measures the main factors that appear separately in other famous questionnaires: Job Content Questionnaire and Effort-Reward Imbalance Questionnaire (both mentioned in the Introduction section). Definitively, this tool allows detecting the factors that precede chronic stress in a profession such as the police, in which personnel are capable of suffering risk situations that generate stress and produce psychological exhaustion [39, 40].

Despite of the strengths of DECORE-21 questionnaire, it would be interesting in future studies to examine other types of validity, discriminating validity for example, as well as to extend the sample and to compare different results with the ones obtained in other countries. All these studies can improve the assessment of occupational stress in police officers.
